# Supplementary material for: Examining the Negative Sentiments Related to Influenza Vaccination from 2017 to 2022: An Unsupervised Deep Learning Analysis of 261,613 Twitter Posts
Source: Vaccines (Basel). 2023 May 23;11(6):1018. doi: 10.3390/vaccines11061018 (PMC10305179; doi:10.3390/vaccines11061018)
Supplement: Supplementary file 1 [file vaccines-11-01018-s001.zip › vaccines-2355865-supplementary.pdf]

Table S1. Sample tweets for the various topics, as per BERTopic modelling

S/N	Tweet
Topic 1 (*effective, like flu vaccine, arm, gp, day, nhs, strain, flu, flu shot)*	
1.	If people are mad at people attending a golfing dinner, they will be riots when people realise how many people don't get the flu vaccine putting everyone from babies to the elderly at risk.
2.	What is your problem , what happened to free will ? If some people don t want it then why should we force them ? Not everyone gets the flu jab every year
3.	What about the new flu vaccine they have to come out with every year when the strain mutates? I know it isn t mandatory but it s still a new vaccine every year. I m not for mandatory vax but there s some wild anti vax stuff out there
4.	Just had breaking news here in my state where ALL children are going to be required to have the """" flu vaccine"""" before heading back to school. People are pissed as they should be. NO ONE is going to make me get any flu shot if I don't want one ever. I'll move to another country
5.	There s no sound data to support use of the flu vaccine therefore it should be made available but not mandated. They of all people should be able to determine whether they should take the shot or not.
6.	The flu vaccine is not mandated, nor has the flu ever completely changed our way of life. Why hasn't the flu vaccine been mandated? Not enough people dead? Thats sick
7.	Are you forced to get the H1N1 flu vaccine or Sars vaccine? How about the shingles vaccine or the bird flu vaccine ? Forced is the key word here! Companies charging you more for health insurance because your well & won't take vaccine!! WRONG IN EVERY WAY!!!
8.	They've never coerced everyone into the flu vaccine. Let's hope they don't start now. Reality is stranger than fiction at the moment.
9.	How 'bout we start mandating weekly testing for folks who won't take the flu vaccine, too? Why doesn't gov't insist we take flu vaccine, too? SMH People just love tyranny!
10.	If the vaccine stopped someone from contracting it and spreading Covid I think you would have close to 100% mandate compliance. Unfortunately we re not there yet so to mandate it and fire people for somthing that basically works like a flu vaccine is sad.
11.	You're forgetting that we have a Flu vaccine that isn't pushed in the same way, and no one is excluded from society for not having it. It's not even freely available to all unless you pay for it. We should not EVER support proof of vaccination to gain entry anywhere. Absurd!!!

12.	where exactly do you draw the line? I'm not anti vax, but I don't want Covid vaccine and not interested in flu vaccine either. You can't make some vaccines mandatory and others not.
13.	This is profoundly different. The flu jab protects you and it s your risk - no problem. Now people want to mandate that risk to everyone else as well even if they are confident in their own immunity. In doing this we deny humanity s right to a strong natural immune system.
14.	It s a personal choice to take an untested vaccine. As is smoking, or how much food you put in your face. Using your logic you ll force everyone to get a seasonal flu jab too, because approx 80k end up in hospital yearly.
15.	So if we don't get the flu vaccine or the pneumonia vaccine we shouldn't be able to go to the hospital we should just stay at home and die instead of going to the hospital and trying to save our lives listen to how you sound
16.	So does this mean they have never agreed to have any vaccines, ever? I ve never heard of people rejecting the flu vaccine or say, typhoid vaccine if they re travelling to affected counties. Who is delivering this message to ordinary families?
17.	People who choose not to vaccinate understand that they may contract the illness. Does it matter if it is from someone vaccinated or a person who is infected naturally? I believe that natural immunity is better than vaccine immunity. That is why I don t get the flu vaccine.
18.	Flu strains the healthcare industry as well but you don t regulate the flu vaccine. Sounds like you be a better healthcare system
19.	Can't force people to have a vaccine! You can't force people to get the flu vaccine and you've never worried about that
20.	Also, unvaccinated people can prolong the pandemic since they can overwhelm hospitals requiring vaccinated people to social distance and wear masks. Taking away the freedoms of everyone else. I don't think the MMR or Flu Vaccine should be mandatory but Covid vaccine should be.
Topic 2 (*trump, vaccine work, flu vaccine work, president, guy, thinks, solid, solid flu vaccine, solid flu, corona*)	
1.	The one.truly good thing Trump has supported is the promising vaccines being manufactured while being evaluated. The other thing he should be doing is talking up taking the vaccine,.as well as the flu vaccine..
2.	He seems to be oblivious to the fact that far more people die of covid and there is actually a flu vaccine.

3.	you know what he meant lugs, nobody pushing the flu vaccine at every breathing human, offering all sorts of incentives and shutting down the freedom to travel, freedom to work,etc
4.	#TimothyGoneGone Feb.12th. 2018 He loves his foundtRump CDC= Right wing =Anti-Scientist- Vaccines-Etc. Timothy is A Scientist at the CDC I hope he did not find out tRump/Pence is """"Doctoring"""" Flu Vaccine as a means of banning All Vaccines
5.	He's recently made negative comments about the flu vaccine
6.	Heard him say that at the rally, but heard a radio interview from last week where he said he didn't. Never takes flu vaccine either.
7.	I m not sure Bill believes the covid vaccine works given his previous advocacy agaisnt the flu vaccine
8.	Yes, it really made me mad! And how he goes on about how fast he made it available. I'll bet he did NOT take the vaccine - he just commented that he doesn't take them...ever..never any flu vaccine. What is going on? He pushes it every time he speaks!
9.	I'm guessing his ignorant ass took a """"one size fits all"""" polio vaccine. How about the flu vaccine? Measles, mumps, rubella, tetanus, pneumonia, shingles? 'That's crazy': Democrat shuts down Bill Maher's rant against vaccine booster shots on 'Real Time'
10.	Being mad at Beto is like being upset that a company raised $800 million for cancer research but won t share it with flu vaccine researchers....the importance of one outweighs the other sometimes.....
11.	Hold on ! He was goin OTT about covid Not being like Flu a while ago. So by his reckoning we will hv lockdown every flu season. Flu jab only 50% effective in lessoning symptoms - covid jab MUCH more effective. 2018 saw + 50,000 UK flu deaths & no-one batted eyelid.
12.	Questioned the swine flu jab, ignored the serious adverse reactions and deaths following experimental covid jabs, he lost his way.
13.	You re really putting your faith in a man who doesn t believe in medicine or science and opts for homeopathy, refuses the flu jab and clearly hasn t had and won t have the Covid vaccine. Of course he d have led us better during a pandemic, .
14.	I'd love to hear how Vichy French would explain natural immunity? I'd also ask him why he isn't pushing for a flu vaccine?
15.	Of course it worked, but you wouldn t understand of course, he s concerned about variants brought into the country, after all flu vaccine doesn t last forever and has to be renewed every year and that is less virulent than Covid.
16.	Tuesday October 16 - NBC Nightly News: Trump: Prince """"totally denied"""" knowledge of missing journalist; Hurricane death toll soars to 29; Rare access inside flu vaccine lab; Mega Millions mania Via

17.	Wrong. Problem is covid vaccines were, out of necessity, rushed out, with minimal testing. Trump gets credit for working with Pharma to meet the timeline, but any credible medical researcher knows the risks involved. Standard flu vaccine went through the complete FDA protocol.
18.	The only grain of truth in his positions on this is the part where liberals are unwilling to even consider the possibility that there could be any problems with a new vaccine. The Swine Flu vaccine in the 70s went thru the whole process & still had to be recalled. It happens.
19.	My shock is from when he says he never had flu vaccine, hence I ask if as a politician he never traveled to countries where they are mandatory. I was not focused on this one when I made comment
20.	BERNIE TALKING SHIT ABOUT STATES THAT REFUSE TO FOLLOW NEW WORLD ORDER COVID = FLU VACCINE HAPPY PROPAGANDA..... HE'S ANOTHER PUPPET WHOM NEEDS TO BE REMOVED..... FUCK UR VACCINE AND UR MASK THAT WILL LEAD TO HEALTH COMPLICATIONS..... #FURMARKOFTHEBEAST #FTHENEWWORLDORDER
Topic 3 (*mask, masks, wear, wearing, wear mask, wearing masks, wearing mask, mask flu, wear masks, flu season)*	
1.	Swine flu vaccine was a disaster. Cloth masks are basically ineffective.
2.	Maybe the thinking is why get a flu vaccine when covid protocols and hygiene have all but eradicated it? Unlike covid, its surface transmissible .6ft n masks That said, mask compliance is horrible lately here in roc So i got the flu vax
3.	If masks can save us from covid, they should be able to save us from flu. So why would anyone want a flu jab considering masks are mandatory??
4.	Then i had better use the money that would have paid for my flu jab to buy more masks. You cant have it both ways. #covidvariant #OmicronVariant
5.	I cant afford to pay for a flu jab and pay out for more masks You cant have it both ways.
6.	Now we know that wearing masks will last beyond the vaccines. Also pay out for more masks or pay for flu jab. Ooh choices choices #covidvariant
7.	According to the data only 6 severe cases of the influenza since January 2020. We ve had flu vaccine for decades and similar numbers, so is it that masks and distancing works for influenza but not covid? Influenza is always mentioned every year. 2017-2018 was fairly bad.
8.	Yep many years ago the US Army conducted an experiment that concluded if a person was vaccinated with a live flu vaccine and wore

	mask prolonged afterward they would be more susceptible to developing the flu. Mechanism was increased viral lode from the mask.
9.	Might also be a confounding variable. Healthcare providers who decline the flu vaccine may have certain antiscience beliefs. Those may be translated to antiscience beliefs about masks and even PPE in general. Not saying that's the cause, but that's one possibility.
10.	People are free to not wear one. Just not in public. Millions take a flu vaccine every hear and take every precaution. Covid is NOT a flu. Is is much worse
11.	Last year my father was undergoing immunotherapy. He couldn t have a flu jab. He couldn t have a covid jab. He also wasn t allowed to drive. He had to use public transport. Any bug picked up could have been fatal. That s way I believe face coverings should be compulsory
12.	Aveline: I presume from now on you will be getting the annual flu jab, socially distancing & wearing a mask to save the lives of thousands of people who die each year from flu? If you don't someone might accuse you of spreading flu virus & potentially killing vulnerable people.
13.	Masks do not protect you from a virus. People get the Flu vaccine every year and STILL get the Flu. Let that sink in.
14.	I am not bothering with the flu vaccine the masks killed it last year.
15.	Fauci even said the vaccine will be at best 40% effective which makes sense when you think of the yearly flu vaccine... Mask mandates are fine but people in my area are already stopping the use of them. If Our kids aren't allowed back in school soon, their future is ruined.
16.	When I was waiting for my flu jab last week, the woman next to me showed me a little plastic gadget which she wears inside her mask to keep it off her nose and mouth. Like a little open basket. Â£1.99 in a shop near me, but I haven't tried one myself.
17.	I m double vaccinated, had the flu jab, will get the booster next month. I wear masks indoors if I feel it necessary. Nothing tells me they work. But the most importing is , I believe people have a right to choose whether they wear masks or get vaccinated.
18.	Wish someone would convince the narrow-minded muzzle-obsessed panic-mongered yokels hereabouts of that! They won't give a 'flu jab (even outside!) if I don't wear a face covering and some knob expects everyone at the War Memorial on 8th Nov. to be muzzled too. I despair!
19.	Hmm well if they don't want to wear a mask I dount that want that shit pumped in them anyway. Wether I weara mask or not, I will not be taking the vaccine. Flu vaccine as well as others haven't done shit so

	why would this one. Me and my youngest daughter both got the flu vaccine
20.	I didn t realize that flu vaccine was only 40-50% effective, that does change things a lot. I did look up some flu stats, it s much less deadly than covid-19 (but still does serious damage. I m also uninformed on how flu spreads. Would masks help?
Topic 4 (*asthma, asthmatics, group, asthmaticsatrisk, jab list, flu jab list, priority, jcvi, eligible, asthmatic*)	
1.	Hi, please will you look into the JCVI decision to exclude asthmatics who get the flu jab and take steroid inhalers from the Covid vaccine priority groups? The told in December we'd be in group 6, but have not added us in subsequent updates.
2.	Please could you take this down? Whilst those of us who are very aware of the #asthmaticsatrisk issue are a cynical bunch, this tweet does create false hope for some, as we all get the flu jab yet have been excluded from the Covid jab and this remains the case.
3.	I have Asthma take steroid inhalers 2/3 times daily, have flu vaccine every year and told to be extra careful and even shield due to COVID - apparently though not critical for COVID-19 Vaccine despite suffering from a lung condition? really! Priority group 9!
4.	please try and make someone see sense! All asthmatics entitled to the annual flu vaccine should be group 6! Been told for last year I'm CV and now suddenly in the last week oh your not anymore!!! Fuming is an understatement
5.	Can you link to where it said officially those who receive a flu jab will be in the priority group please as that is relevant to us? & the worse asthmatics who had to shield are covered in group 4 ie if you had a shielding letter you should already have been called.
6.	I don t understand how asthmatics are urged to get a flu vaccine but are now not deemed vulnerable unless they fulfil a fairly restrictive criteria
7.	It is disgraceful that the Government has backtracked on early covid vaccination to people who are offered the flu jab. All asthmatics on steroids were in phase 6, now many are moved to 10. All people classed as vulnerable to covid should be in phase 6.
8.	This is not the fault of asthma UK. I've been steroid controlled (inhaler) for 30 years, missed 1 flu jab, got flu and nearly died. I was younger and fitter then. I don't fancy my chances with Covid and am seething with this decision, but it's not the fault of asthma UK.
9.	On BBC breakfast this morning they've said everyone who has an annual flu jab will get a covid booster, and these people are clinically

	extremely vulnerable. Except many asthmatics who get an annual flu jab, including myself, don't qualify for a covid vaccine yet....
10.	I wonder if that's Vulnerable as per the flu jab list for people with #asthma, or Vulnerable as per the made-up criteria that eliminated 1.6m #asthmaticsatrisk from the Covid priority list...? Sadly I expect it will be the latter. #VaccinesForAsthmatics #FluJabEqualsCovidJab
11.	What s more, now they plan to roll out boosters and flu jab at same time, non Covid priority asthmatics are hardly going to be top of their list for flu jabs. Entirely plausible given the disdain with which asthmatics have been treated #AsthmaticsAtRisk
12.	I am young, I am asthmatic. I was asked in 2020 to take the flu jab due to the risk of Covid-19 combined with the risk of flu. I'd never been asked to before. Why am I not being put at high risk on the covid vaccine list - which is a severe flu? #VaccinesForAsthmatics
13.	#AsthmaticsAtRisk Did you know that asthmatics are the only people always offered the flu vaccine who are NOT being offered the covid vaccine? Yet asthma is respiratory disease & Covid is a respiratory virus.
14.	#asthmaticsunder50 the only fair was flu jab equals Covid jab!
15.	What you say is not happening in reality. My husband is asthmatic, a key worker and at 46 has been told he cannot have covid vaccination as not in priority group. Why is he offered a flu jab annually but not a covid vaccination as a dangerous respiratory virus?
16.	why when it is proven that flu jab eligible asthmatics are at increased risk of hospitalisation, critical care and long covid (I.e. serious disease) and have been classed as clinically vulnerable throughout the pandemic are many being denied their vaccination in group 6?
17.	#isitok that has been vaccinated for his asthma when millions of other asthma sufferers who get the flu jab every year have to wait till summer?
18.	I am so gutted - after a year of being told I am clinically vulnerable as a long term asthma sufferer (steroid inhalers, annual flu jab) I am now told I won t get a vaccine until autumn.
19.	People with #asthma are at risk so should get a flu jab. People with asthma are at risk but won't get a #covidjab. The dissonance is astounding. I won't be economically participating when #Lockdown3 ends, I'll be staying at home, because I won't be vaccinated and am at risk.
20.	So Asthma is serious enough to need an annual flu jab, but not serious enough to need the covid jab? In all the absolute sincerity I can muster, I fucking hate Torys. Genuinely hope they all suddenly struggle to simply breathe.

Topic 5 (*mrna, mrna flu, mrna flu vaccine, vaccine mrna, mrna vaccines, technology, mrna vaccine, flu vaccine mrna, rna, moderna*)	
1.	$MRNA -13% UPDATED: Moderna says its mRNA flu vaccine cleared early studies, but comparisons with other vaccines drive stock down
2.	MRNA flu vaccine in the works now as well. Roll on sudden deaths
3.	Whichever way smarm manifests itself David Mellor exudes buckets of it. The flu vaccine and Covid therapy are 2 different things. Check out MRnA. I ve had the jab old boy so you must If it looks like a jerk it therefore proves
4.	Also, there's a big difference between getting the flu jab, which has been through rigorous trials, testing, peer-review, and risks now known to us, than there is when taking this new """"RNA-treatment"""", which its risks are completely unknown to us long-term.
5.	It should have been obvious when they kept telling ppl to keep getting jabbed that the jabs weren't working. And there has been a non mRna vaccine available for over a yr thats a traditional vaccine which means a vaccine that's made from the virus itself. Same as a flu vaccine.
6.	In principle, the RNA vaccines should also induce T-cell immunity, but yes, only against one antigen. And live attenuated flu vaccine which should be better at cellular immunity is no more effective than killed vaccine.
7.	I don't know what you're referring to when you say """"The flu vaccine took 27 years, and the one you gave to your kid took a year (ish) and it s fundamentally different than any other human vaccine."""". mRNA vaccines have been in development for decades.
8.	Nothing sexy about a 2 dollar flu jab though is there? Why we are pushing mRNA is beyond me.
9.	The flu vaccine has has been around since the 30's (in response to your tweet about the flu vaccines and knowing what we're getting in to. mRNA have been around for 2 decades but wasn't talking about that. Maybe read your own tweet first, that was your topic. If the last time...
10.	How does the flu vaccine/childhood vaccines etc differ from the EUA mRNA nano technology? They are two different delivery systems. If they are not - how are they the same? Is there a small portion of Corona virus in the mRNA shots? I m willing to learn more.
11.	Last time I checked the flu vaccine wasn't an mRNA experimental treatment and wasn't required to partecipate in society
12.	I heard that they were putting MRNA into the flu jab this year
13.	That would provide decades of long term safety data on that technology prior to its use in the flu vaccine. Therefore you're comparing apples to oranges. mRNA is a brand new technology that was never utilized in any approved vaccine prior to COVID. 2

14.	Oh the old compare too, of the under-utilized brain,. Hello, there is a difference between the two, the flu vaccine and every OTHER vaccine you choose as your comparison, apples to oranges when compared to MRNA. Either educate or stifle, it is pretty simple .
15.	So sad.... and criminal! Apparently, the flu jab is now mRNA.
16.	The flu vaccine uses 50-year-old technology. We should replace it with an mRNA vaccine, which will be far more effective
17.	It s a reputable name in the vaccine world and is more like the existing flu jab, I m not sold on the mRNA vaccines, and for the record I m by no means an anti vaxer!
18.	Vaccinations have also led to the resurgence of serious diseases (vaccine-derived polio) and late effects such as narcolepsy induced by Pandemrix swine flu vaccine. We know nothing about the long-term consequences of mRNA vaccines so hesitancy is sensible
19.	mRNA flu vaccines are in Phase III from the majors. What's their obsession with modifying every American's DNA?
20.	You claimed it was """"literally synthetic messenger RNA."""" I found what you are trying to hide based on the report that two amino acid substitutions ended the 1918 influenza vaccine and the patent for light-activated carbon fixation (RNA interference).
